# Well Leg Compartment Syndrome after Contralateral Femoral Neck ORIF

**DOI:** 10.1155/2020/8859954

**Published:** 2020-07-30

**Authors:** Christopher Shultz, Kristen Baca, Michael Decker

**Affiliations:** ^1^The University of New Mexico Department of Orthopaedics and Rehabilitation, MSC 10 5600 1 University of New Mexico, Albuquerque, NM 87131, USA; ^2^The University of New Mexico College of Medicine, 915 Camino de Salud NE, Albuquerque, NM 87106, USA

## Abstract

The authors present a case of WLCS after femoral neck fracture fixation. While this is a rare complication, a high index of suspicion should exist. Surgeons should use well leg holders with caution and limit utilization time. Alternative methods of positioning to allow for fluoroscopic imaging exist. WLCS remains a clinical diagnosis; intracompartmental measurements can be used but should be cautiously interpreted. When the diagnosis of WLCS is made, emergent fasciotomies of the affected compartments should be performed. Surgeons should be aware of this complication when using a well leg holder and the potential catastrophic consequences if left ignored.

## 1. Introduction

Well leg compartment syndrome (WLCS) is a serious surgical complication that has been documented in a variety of surgical procedures [1]. WLCS is defined as an acute lower limb compartment syndrome developing without trauma or preexisting vascular disease (BJS guidelines) [2]. It is associated with prolonged operative positioning with the hips and knees flexed, especially in the Trendelenburg position [2]. WLCS incidence is estimated to be 1/3500 surgeries utilizing a well leg holder; however, there is speculation that it is underdocumented due to a range of symptom severity and misdiagnosis as a deep vein thrombosis [3]. Multiple risk factors exist for WLCS, and early diagnosis is imperative as it constitutes a surgical emergency. WLCS occurs when external pressure is placed on the well leg during surgical procedures, due to either the hemilithotomy or the lithotomy leg positions. Prolonged surgical procedures in these positions can cause ischemia and edema within the leg compartments, followed by reperfusion injury once the leg is released from the compromising position. As the compartmental pressures increase, muscle necrosis and hypoxia occur, leading to loss of leg function, kidney failure, or death [4]. WLCS presents as leg pain that cannot be addressed with pain medications and is usually noticed by the patient within the first 24 hours after surgery. Pain on passive movement at the ankle joint and elevated creatinine kinase levels support a diagnosis of WLCS [2]. In order to prevent further muscle damage, emergent fasciotomies to decompress the affected compartments are essential.

## 2. Statement of Informed Consent

This patient has been informed that data concerning her case has been submitted for publication, and she happily agrees.

## 3. Case Report

**Figure 1:**
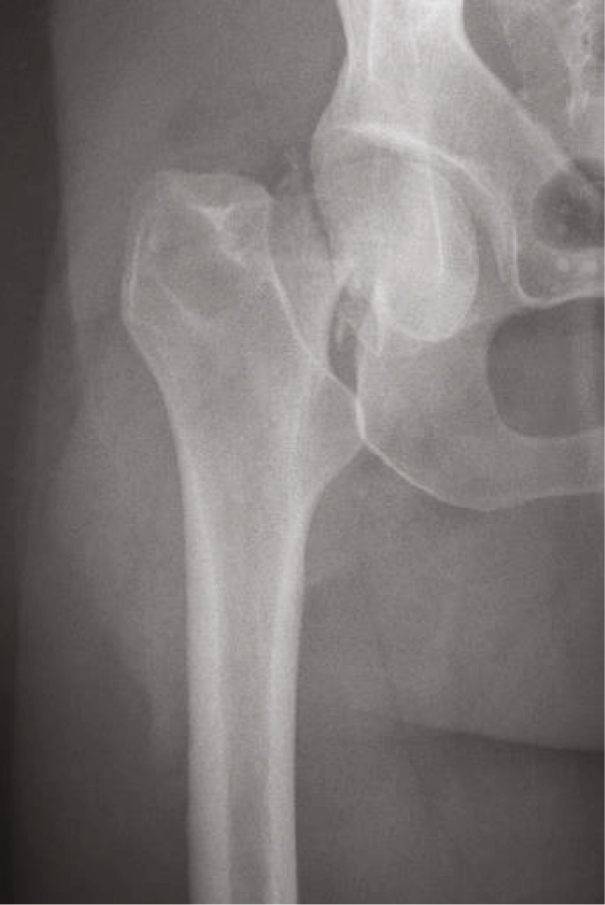
AP radiograph of the right hip demonstrating a subcapital femoral neck fracture.

CP is a 42-year-old female who presented to our institution after falling from a tree complaining only of right hip pain. Physical exam revealed palpable pedal pulses in the bilateral lower extremities and normal sensorimotor examination in the femoral and sciatic nerve distributions. Pain was elicited with internal and external rotation of her right hip. No paresthesias, swelling, or pain with passive stretch of the bilateral lower extremities was noted. Radiographs revealed a comminuted subcapital femoral neck fracture (Figure 1). No other injuries were identified on secondary examination.

**Figure 2:**
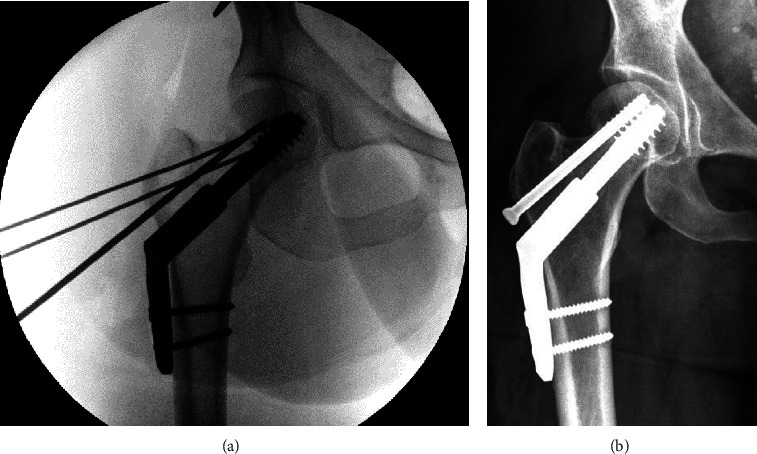
(a) Intraoperative right hip AP fluoroscopic image demonstrating reduction and implant placement with provisional fixation still in place; (b) final AP radiograph of the right hip demonstrating healed subcapital femoral neck fracture at 6 months postoperatively.

The patient was taken to the operating room approximately 11 hours after injury. She was positioned supine on an OSI flat top table with the left leg in a well-padded well leg holder, positioning the knee and hip at 45 degrees of flexion to facilitate fluoroscopic imaging. A sequential compression device (SCD) was placed on the noninjured calf. Open reduction was carried out through a Smith-Peterson approach. A lateral approach to the femur was utilized for provisional fixation and placement of a 135-degree hip screw side plate and a 7.0 mm partially threaded cannulated screw (Figure 2(a)). Upon fluoroscopic confirmation of reduction and final implant placement, the well leg was placed flat on the operative table by the circulating nurse. Surgical wounds were closed, and the patient was transferred into the recovery area. Total time with the well leg elevated was 109 minutes.

While recovering, the patient reported new anterior leg pain and paresthesias over the dorsal foot of the well leg. On exam, she was found to have diminished sensation over the dorsal foot, pain with passive flexion of the ankle and great toe, and edema isolated to the anterior and lateral compartments of the leg. She had no pain with passive dorsiflexion of the ankle and great toe and no pain with palpation of the deep and superficial posterior leg compartments. The well leg was elevated, ice applied, and the patient was closely monitored over the course of an hour. Over this time period, her pain continued to worsen despite increasing doses of opioid analgesia and ketorolac.

The diagnosis of compartment syndrome was made, and the patient returned to the OR for selective fasciotomies of the anterior and lateral compartments. The muscle of the anterior and lateral compartments was mildly edematous, contractile, and bleeding. The skin incision was closed with a suture to allow for quick suture removal should muscle swelling worsen. The patient reported immediate improvement of her pain and paresthesias.

The remaining postoperative course was uneventful. She was able to bear weight on her left leg following fasciotomies and went on to an uneventful recovery of her femoral neck fracture. On the follow-up at 6 months postoperatively, she reported no hip pain with normal sensation in both legs and a healed fracture (Figure 2(b)).

## 4. Discussion

**Figure 3:**
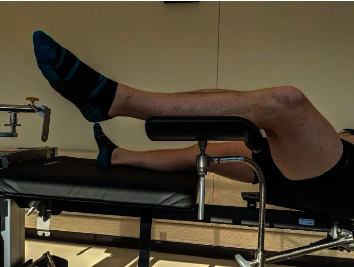
Demonstration of a Gopel style well leg holder on a patient in the supine position.

Well leg compartment syndrome is uncommon, despite the frequency of lower extremity and pelvic surgical procedures performed annually [3]. The low incidence of WLCS makes identification and prevention challenging. The distinguishing factor of WLCS is the seemingly innocuous exposure in a well leg holder, such as the Gopel support (Figure 3), which should be avoided if possible. The utilization of a well leg holder allows for improved fluoroscopic imaging during surgery. However, other strategies can be utilized to facilitate intraoperative imaging. Brouze et al. described securing the nonoperative leg to the traction bar of a fracture table with cushions [5]. Alternatively, the nonoperative leg can be prepped into the surgical field and a sterile drape applied to the well leg to allow for well leg positioning only during use of fluoroscopy.

When a well leg must be employed, the surgeon must be aware of potential risk factors for WLCS. Current literature varies greatly regarding a time frame during which WLCS develops. Tan et al. found that intracompartmental pressures rose from 9 mmHg to 27 mmHg with placement of the nonoperative leg in the well holder and continued to rise by 1.1 mmHg/hour throughout the procedure [6]. Meyer et al. found that anterior compartmental pressure increased from 13 to 25.8 mmHg when placing the leg from the supine position into a calf-supporting well holder [1]. The consensus amongst most authors is that the well leg should be relaxed and lowered down every 2 hours, similar to the well-accepted timing of deflating a tourniquet. Monitoring and maintenance of systemic perfusion pressure may minimize risk of WLCS. The objective measure of Δ*P*_*D*_ for diagnosis of compartment syndrome has been well accepted, with Δ*P*_*D*_ ≤ 30 mmHg being highly associated with clinical compartment syndrome in 3 of 116 patients prospectively monitored with tibia fractures [7, 8]. Based on the findings of Tan and Meyer, it appears that a goal intraoperative diastolic blood pressure ≥ 60 mmHg would be a safe benchmark.

BMI, age, and gender have been identified as risk factors that must be considered perioperatively. BMI ≥ 25 has been associated with increased risk of WLCS due to a decrease in ankle blood pressure and increased intramuscular pressure when in the hemilithotomy position [2]. Younger, male patients are also at increased risk, potentially due to increased muscle mass and overall stronger fascia which can maintain elevated compartmental pressures [9].

When WLCS is suspected, confirming the diagnosis can be challenging, as the decision for a return to the operating room hinges on prompt identification. The diagnosis remains clinical, with the most salient feature being worsening leg pain despite appropriate leg elevation and analgesic medication. Other clinical features such as paresthesias, pulseless extremity, and pallor may also be suggestive of compartment syndrome but are not required for the diagnosis. When the diagnosis of WLCS is unclear, intracompartmental pressures may be measured but should be utilized with caution. In a prospective review of 48 patients with surgically treated tibial shaft fractures without clinical signs of CS, intraoperative compartmental pressure measurements revealed a 35% false-positive rate [10]. Other authors express similar concern in the specificity of one-time intracompartmental pressure monitoring for detecting acute compartment syndrome and the possibility of unnecessary fasciotomies [11]. Continuous compartment monitoring may be more useful in confirming the diagnosis. With this method, McQueen et al. analyzed 850 patients with diaphyseal tibia shaft fractures and reported a specificity of 98% and a sensitivity of 94% for detecting acute compartment syndrome [8]. For the authors, experienced intracompartmental pressure measurement should be limited to patients in which a physical assessment of pain cannot be obtained (AMS, intubation/sedation, etc.). Compartmental pressure measurements should never obviate a concerning physical exam.

When WLCS is diagnosed, fasciotomies should be performed emergently. In cases where only one or two compartments are involved, selective fasciotomies may be considered. In a review of 51 patients diagnosed with acute compartment syndrome in the setting of a tibial shaft fracture, Hatz et al. found that 67% of patients were able to safely undergo selective compartment fasciotomies (the remaining 33% required four compartment fasciotomies) [12]. It is worth noting that in all cases the anterior compartment was involved. There remains a paucity of data regarding safety of selective fasciotomies in cases of WLCS; in the current case presented, only the anterior and lateral compartments were affected based on physical exam and therefore were the only compartments released. Our patient had no physical exam findings of compartment syndrome in the posterior compartments. This remained the case postoperatively and at the final follow-up. In cases where all four compartments are suspected, single and two incision fasciotomies are both safe options and should be left to the surgeon's discretion and experience with each procedure [13].
